# Downregulation of the Repressor Element 1-Silencing Transcription Factor (REST) Is Associated with Akt-mTOR and Wnt-β-Catenin Signaling in Prion Diseases Models

**DOI:** 10.3389/fnmol.2017.00128

**Published:** 2017-05-03

**Authors:** Zhiqi Song, Syed Z. A. Shah, Wei Yang, Haodi Dong, Lifeng Yang, Xiangmei Zhou, Deming Zhao

**Affiliations:** The State Key Laboratories for Agrobiotechnology, Key Lab of Animal Epidemiology and Zoonosis, Ministry of Agriculture, National Animal Transmissible Spongiform Encephalopathy Laboratory, College of Veterinary Medicine, China Agricultural UniversityBeijing, China

**Keywords:** RE1-silencing transcription factor, prion diseases, neuroprotective mechanism, the Akt–mTOR signaling, the Wnt-β-catenin signaling

## Abstract

Prion diseases are a group of infectious diseases characterized by multiple neuropathological changes, yet the mechanisms that preserve function and protect against prion-associated neurodegeneration are still unclear. We previously reported that the repressor element 1-silencing transcription factor (REST) alleviates neurotoxic prion peptide (PrP106-126)-induced toxicity in primary neurons. Here we confirmed the findings of the *in vitro* model in 263K infected hamsters, an *in vivo* model of prion diseases and further showed the relationships between REST and related signaling pathways. REST was depleted from the nucleus in prion infected brains and taken up by autophagosomes in the cytoplasm, co-localizing with LC3-II. Importantly, downregulation of the Akt–mTOR and at least partially inactivation of LRP6-Wnt-β-catenin signaling pathways correlated with the decreased levels of REST *in vivo* in the brain of 263K-infected hamsters and *in vitro* in PrP106-126-treated primary neurons. Overexpression of REST in primary cortical neurons alleviated PrP106-126 peptide-induced neuronal oxidative stress, mitochondrial damage and partly inhibition of the LRP6-Wnt-β-catenin and Akt–mTOR signaling. Based on our findings, a model of REST-mediated neuroprotection in prion infected animals is proposed, with Akt–mTOR and Wnt-β-catenin signaling as the key pathways. REST-mediated neuronal survival signaling could be explored as a viable therapeutic target for prion diseases and related neurodegenerative diseases.

## Introduction

Prion is a protein-conformation-based infectious agent (Zhang et al., 2014; Wang et al., 2015) and causes transmissible spongiform encephalopathies (TSEs) in animals and humans (Wadsworth and Collinge, 2011). The neuropathology of prion diseases is characterized by synaptic alterations, spongiform degeneration, neuronal cell death, reactive gliosis, and amyloid plaque formation (Soto and Satani, 2011; Song et al., 2013). Our recent study indicated that the transcription factor REST, a RE1-silencing transcription factor may function as a neuroprotector in prion diseases (Song et al., 2016). REST, also known as neuron-restrictive silencer factor (NRSF) (Schoenherr and Anderson, 1995), is a transcriptional regulator, and with other factors, coordinately regulates multiple aspects of neurogenesis, orchestrates neural differentiation, and preserves the unique neural phenotype (Song et al., 2015). Perturbation of REST expression or function results in early embryonic lethality (Chen et al., 1998) and ectopic expression of neuronal genes in non-neuronal tissues (Ballas et al., 2005). REST protein also protects aging neurons from death by repressing genes that promote cell death and Alzheimer’s disease (AD) pathology and inducing the expression of stress response genes (Lu et al., 2014). REST alleviates neurotoxic prion peptide (PrP106-126)-induced synaptic abnormalities, neurofibrillary degeneration and neuronal death partially via the low-density lipoprotein receptor-related protein 6 (LRP6)-mediated Wnt-β-catenin signaling in primary cortical neurons (Song et al., 2016). Overexpression of REST protects neurons through upregulated expression of postsynaptic protein, stabilized level of pro-survival protein and suppressed pro-apoptotic protein (Song et al., 2016).

There are still many questions remain to be answered regarding the role of REST and mechanisms in neuroprotection. Firstly, if REST acts as a neuroprotective regulator in prion diseases, what is the pattern of expression and distribution of REST in the brain of scrapie-infected experimental animals? Secondly, what is the relationship between REST and autophagy system and associated signaling pathway *in vivo*? Autophagic vacuoles have been observed in the neurons of experimental hamsters with prion diseases, as well as in synapses of human Creutzfeldt-Jakob disease (CJD) and Fatal familial insomnia (FFI) patients (Boellaard et al., 1991; Jeffrey et al., 1995; Liberski et al., 2004). We also found that PrP106-126 induces structural pathology in primary cultured cortical neurons (PCCN) including myelin figures or concentric lamella structures and autophagosomes or autophagolysosomes (Song et al., 2016). Overexpression of REST protects PCCN cells from PrP106-126-induced morphological changes and reduces the formation of autophagic vacuoles (Song et al., 2016). Knockdown of REST disrupted the mTOR signaling pathway, which is the most important negative regulator of autophagy. Silence of REST reduced cell viability in the human oral squamous cell carcinoma (SCC) cells in a time-dependent manner, associated with the activation of apoptosis elements and DNA fragmentation (Cho et al., 2014). Therefore, we further asked what the role of REST in the Akt-mTOR pathway is in prion diseases.

The Wnt-β-catenin signaling pathway has long been associated with the modulation of neurogenesis, dendritic morphogenesis, and synaptic function (Inestrosa and Varela-Nallar, 2014, 2015). Wnt ligands activate the pathway by binding to LRP6 together with Frizzled receptors and transduce signals through the stabilization of β-catenin. During normal aging (Lu et al., 2014) or in chick spinal cord (Nishihara et al., 2003), REST is induced in part by cell non-autonomous Wnt signaling. However, both the Wnt signaling and the REST induction of patients with AD are suppressed in, leading to neurodegeneration (Lu et al., 2014). Our previous study provides the first observation that LRP6 evidently regulates the expression of REST in addition to β-catenin (Song et al., 2016). Moreover, in the Wnt pathway, there are several genes influence NRSF transcription (Nishihara et al., 2003; Kaneko et al., 2014; Song et al., 2015). In this respect, REST is at least in the downstream of LRP6 in the Wnt signaling pathway. Additionally, previous studies also demonstrated that REST colocalized with β-catenin in aging brain (Lu et al., 2014) or primary cortical neurons (Song et al., 2016). In PC12 cells, raised expression of REST correlates with nuclear accumulation and co-transcriptional activation of β-catenin (Tomasoni et al., 2011). These researches unveil the cooperative relationship of REST with β-catenin in the Wnt signaling. REST plays a strategic role not only in neurogenesis but also in neurodegeneration (Song et al., 2015). Therefore we further explored the potential function and signal loop of REST to related factors in the Wnt signaling *in vivo* and *in vitro*. What’s more, β-catenin as the indicator of the activation of Wnt-β-catenin signaling, and GSK3β as the marker of inhibition of the same signaling pathway were unaffected after prolonged exposure of PCCN to the prion peptide (Song et al., 2016), but the activated form of these molecules has not been investigated.

Herein, we studied the expression and distribution of REST in scrapie-infected hamsters, and REST expression and associated changes of the Akt-mTOR Wnt-β-catenin signaling pathways. We observed downregulation of REST, the inhibition of autophagy system-associated Akt-mTOR signaling pathway and the partially inactivation of the Wnt-β-catenin signaling pathway in the *in vitro* and *in vivo* prion disease models. Furthermore, overexpression of REST alleviated prion peptide-induced inactivation of the Akt-mTOR and Wnt-β-catenin signaling pathways in PCCN. As far as we know, this is the first report that REST is reciprocally related to Wnt signaling. In conclusion, REST plays a critical neuroprotective role partly via restoring the Akt-mTOR and Wnt-β-catenin signaling pathways in prion diseases.

## Materials and Methods

### Animal Ethics Statement

All experimental procedures for the laboratory animals in this study were performed and approved according to the strict guidelines of Chinese Regulations of Laboratory Animals—The Guidelines for the Care of Laboratory Animals (Ministry of Science and Technology of People’s Republic of China) and Laboratory Animal Requirements of Environment and Housing Facilities (GB 14925–2010, National Laboratory Animal Standardization Technical Committee). The license number related to their research protocol was 20110611–01 and the animal study proposal was approved by The Laboratory Animal Ethical Committee of China Agricultural University, Beijing China. We aimed to minimize the suffering of the animals and to reduce the number of animals studied at minimum.

### Animal Model of Prion Diseases

The infectious model of prion disease in hamster was described previously (Shah et al., 2017). This is a very good model for TSE-related studies, as it reproduced many of the clinical, neuropathologic, and biochemical aspects of the disease in humans and other mammals in a relatively short incubation period as compared with other animal models. Five-week-old Syrian golden hamsters (*n* = 30, every group consisted of 15 animals) were used in the current study. Each animal was housed on a separate cage. Syrian Golden Hamsters were injected intraperitoneally (i.p.) with 75 μl of 10% brain homogenate in phosphate buffered saline obtained from terminally ill hamsters infected with the 263K strain of prion according to previous protocols (Bolton, 1998; Xie et al., 2013; Chen et al., 2014; Shah et al., 2017). The animals showed first sign of disease after 70–90 days post infection. The disease was assigned to five stages based on clinical signs (Shah et al., 2017): (1) normal animal; (2) rough coat on limbs with erect hairs; (3) extensive rough coat, hunchback, circling, and visible motor abnormalities; (4) urogenital lesions; and (5) terminal stage of the disease in which the animal presented with cachexia and prostration (with little movement). Ten infected animals were randomly selected at the terminal stage (Stage 5) each time and the brain tissues were separately collected. The brains were removed and frozen at -80°C or fixed in 10% buffered formalin solution. Brains of 10 age matched control hamsters each time were sacrificed as healthy controls.

### Preparations of Brain Homogenates

Frozen brain samples were homogenized in RIPA buffer containing a cocktail of protease inhibitors (Roche, Basel, Switzerland) and were sonicated for 15 s, and then centrifuged at 20,000 *g* for 5 min. The supernatants were collected and boiled for 10 min after addition of a loading buffer (250 mM Tris-HCl pH 6.8, 10% SDS, 0.5% BPB, 50% glycerol, 0.5 M DTT). The protein concentration of the supernatants was measured using the BCA assay (CWBio). Protein extracts (10 μl) were subjected to SDS-PAGE, and western blotting (Shah et al., 2017).

### PrP^Sc^ Detection Assay

The presence and quantity of PrP^Sc^ in brain homogenates of sick animals was measured by a standard assay by determining the resistance of misfolded proteins to degradation by proteinase K. Brain homogenates were incubated in the presence of proteinase K (50 μg/ml) for 1 h with gentle agitation at 37°C. The digestion was stopped by adding electrophoresis sample buffer. Protease-resistant PrP^Sc^ was detected by western blotting, as previously described (Xie et al., 2013; Shah et al., 2017). Briefly, proteins were fractionated by sodium dodecyl sulphate-polyacrylamide gel electrophoresis (SDS-PAGE), and electroblotted onto PVDF membranes (Immobilon-PSQ, ISEQ00010, 0.2 μm). Blots were blocked by 5% skim milk in TBST (25 mM Tris base, 137 mM sodium chloride, 2.7 mM potassium chloride and 0.05% Tween-20, pH7.4) for 1 h at room temperature, then with the 3F4 antibody (SIGNET, SIG-39600, 1:1000, CA, USA) at 4°C overnight and the corresponding HRP-labeled secondary antibody for 50 min at 37°C. The signal was detected using an enhanced chemiluminescence (ECL) detection kit (Bio-Rad, USA).

### Reagents

Rabbit polyclonal anti-REST antibody (07-579) (1:500) was purchased from Millipore. Rabbit polyclonal anti-LRP6 antibody (C5C7) (#2560) (1:500), rabbit monoclonal anti-p70 S6 Kinase antibody (#2708T) (1:500), rabbit monoclonal anti-p-p70 S6 Kinase antibody (Thr389) (108D2) (1:500), rabbit monoclonal anti-4E-BP1 antibody (53H11) (#9644T) (1:500), rabbit monoclonal anti-p-4E-BP1 antibody (Thr37/46) (236B4) (1:500), rabbit monoclonal anti-p-Akt antibody (Ser473) (D9E) (1:500), rabbit monoclonal anti-PRAS40 antibody (D23C7) (#2691T) (1:500), rabbit monoclonal anti-p-PRAS40 antibody (Thr246) (C77D7) (1:500), rabbit monoclonal anti-mTOR antibody (7C10) (1:500), rabbit monoclonal anti-p-mTOR antibody (Ser2448) (1:500), rabbit monoclonal anti-p-GSK-3β antibody (Ser9) (D85E12) (1:500), rabbit polyclonal anti-p-β-Catenin antibody (Ser552) (1:500), rabbit polyclonal anti-p-β-Catenin antibody (Ser33/37) (1:500), rabbit monoclonal anti-CREB antibody (D76D11) (1:500), rabbit monoclonal anti-p-CREB antibody (Ser133) (87G3) (1:500), rabbit monoclonal anti-eIF2α antibody (D7D3) (#5324T) (1:500), rabbit monoclonal anti-p-eIF2α antibody (Ser51) (D9G8) (1:500) and mouse monoclonal anti-HA tag antibody (6E2) (#2367) (IF 1:100) were purchased from Cell Signaling Technology (Danvers, MA, USA). Rabbit polyclonal anti-REST antibody (22242-1-AP) (1:200), mouse monoclonal anti-LC3-II antibody (66139-1-lg) (1:200), rabbit polyclonal anti-Synaptophysin antibody (17785-1-AP) (1:200), rabbit polyclonal anti-PSD-95 antibody (20665-1-AP) (1:200), the rabbit polyclonal anti-Akt antibody (10176-2-AP) (1:200), rabbit polyclonal anti-beta-Catenin antibody (51067-2-AP) (1:200), rabbit polyclonal anti-GSK3β antibody (22104-1-AP) (1:200), mouse monoclonal anti-GAPDH antibody (60004-1-lg) (1:1000), and rabbit polyclonal anti-Lamin B1 antibody (12987-1-AP) (1:500) were purchased from Proteintech Biotechnology (Chicago, IL, USA). Rabbit polyclonal anti-rat β-actin antibody (Cat No.: AP0060) (1:1000), and goat anti-rabbit IgG (H&L)-HRP secondary antibody (Cat No.:BS13278) (1:5000) were purchased from Bioworld Technology (Nanjing, China Bioworld Technology, Inc.). Goat anti-mouse IgG (H&L)-HRP secondary antibody (1:100), TRITC-conjugated AffiniPure Rabbit Anti-Goat IgG (H+L) (ZF-0317) secondary antibody (1:100), Alexa Fluor 594-Conjugated AffiniPure Goat Anti-Rabbit IgG (H+L) (ZF-0516) (1:100), Peroxidase-Conjugated Affinipure goat anti-rabbit IgG (H+L) (ZB-2301) (1:5000) and rabbit anti-goat IgG (H+L) (ZB-2306) (1:5000) were purchased from Beijing ZSGB Biotechnology. Donkey anti-rabbit IgG/FITC (bs-0295D-FITC) (1:100) and Donkey Anti-Goat IgG/Cy3 (bs-0294D-Cy3) (1:100) secondary antibodies were purchased from Beijing Biosynthesis Biotechnology. Neuronal Class III β-Tubulin (Tuj1) antibody (AT809) (1:250), and DAPI dihydrochloride and propidium iodide (PI) were purchased from Beyotime Biotechnology (Wuhan, Hubei, China). Reagents and apparatus used in immunoblotting assays were purchased from Bio-Rad (Richmond, CA, USA).

### Immunohistochemical (IHC) Assays

The hamster brain tissue was fixed in 10% buffered formalin solution, and paraffin sections (5-μm in thickness) were prepared routinely. Sections were treated with an antigen retrieval kit (Boster, AR0022) for 1 min at 37°C and quenched for endogenous peroxidases in 3% H2O2 in methanol for 10 min. After blocking in 1% normal goat serum, the sections were incubated with 1:100-diluted polyclonal antibody for REST at 4°C overnight. Rabbit isotype antibody (Zhongshan Goldenbridge, ZDR-5003) was used as the negative control. Subsequently, the sections were incubated with 1:250-diluted HRP-conjugated goat anti-rabbit secondary antibody (Zhongshan Goldenbridge, ZB-2301) for 60 min, and visualized by incubation with 3,3′-diaminobenzidine tetrahydrochloride (DAB). The slices were counterstained with hematoxylin, dehydrated and mounted on a slide and viewed under an Olympus BX51 microscope. Density analysis was performed using the Image J software (National Institutes of Health, USA). REST staining for each region was separately assessed and the mean optical density (MOD) was recorded. The OD analyses were performed by two investigators in a double-blind manner. Non-specific background correction in each section was done by subtracting the OD value of the blank area obtained from the same section.

### Prion Protein Peptide

The PrP peptide PrP106-126 (sequences KTNMKHMAGAAAAGAVVGGLG), was synthesized by Sangon Bio-Tech (Beijing, China). The purity of prion peptides was >95% according to the data from the synthesizer. FITC-labeled PrP106-126 peptides were purchased from Fanbo Biochemicals (Beijing, China). The peptides were dissolved in 0.1 M PBS to a concentration of 1 mM, and left to aggregate at 37°C for 24 h before each experiment. Experiments were conducted with the final peptide concentration of 200 or 300 μM.

### Primary Cultured Cortical Neurons (PCCN)

Dissociated cerebral cortex neuronal cultures were prepared from postnatal 1-day-old Sprague-Dawley rats, according to the previously described procedure (Song et al., 2014, 2016; Zhu et al., 2015). Briefly, cells were gently dissociated after digestion with papain (Invitrogen). The dissociated cells were plated at a final density of 5 × 10^5^ cells/cm^2^ on polyethyleneimine (Sigma)-coated plates and cultured in DMEM F12 (Hyclone, Logan, UT, USA), supplemented with 2% B27 (Invitrogen) and 0.5% Penicillin-Streptomycin (Gibco). Two days later, 10 μM cytarabine (Sigma) were added to repress the growth of glial cells.

### Plasmids and Transfection

The pCMV-HA-Rest vector of full-length REST (Cat No. PPL50007-2a) was obtained from the Public Protein/Plasmid Library (Nanjing, China, GeneShare Technology, co, Ltd). The pGPH1/GFP/Neo-REST-Rat short-hairpin RNA (shRNA) against Rat REST were obtained from GenePharma (Suzhou, China). Mature antisense sequences of effective shRNAs were 5′-GCTGTGGCTACAATACCAACC-3′ (946-966) (shREST-1) and 5′-GTGCAATTATGTGGCCTCTAA-3′ (1295-1315) (shREST-2). For transfection (Song et al., 2014, 2016; Zhu et al., 2015), cultured primary neurons were washed with Opti-MEM (Invitrogen) in 12-well plates and then transfected with the appropriate plasmids using the Lipofectamine 2000 reagent (Invitrogen) in serum-free Opti-MEM according to the manufacturer’s instructions. The amounts of plasmids and reagents were 2 μg and 3 μl per 12-well, respectively. The culture medium was replaced by the primary cell culture medium 5 h after transfection. Forty-eight hours after transfection, the cells were observed under an upright fluorescence confocal microscope (Olympus, Tokyo, Japan) or subjected to immunoblot analyses.

### Immunofluorescence

For protein localization examination, PCCN grown on cover slips were washed twice with PBS, fixed by Immunol Staining Fix Solution (P0098), blocked 1h at room temperature by Immunol Staining Blocking Buffer (P0102) and then incubated overnight at 4°C with the appropriate primary and secondary antibodies. The nucleus was stained with DAPI. Primary neuronal cells were labeled using murine anti-Tuj1 (neuronal class III tubulin) antibody (Beyotime, China) and fluorescein isothiocyanate (FITC)-conjugated donkey anti-mouse antibodies as the primary and secondary antibodies, respectively.

### Protein Extraction

For the extraction of total cell proteins, primary neurons were washed in PBS after overexpression, knockdown or peptide treatment, and then dissolved and homogenized with pre-chilled RIPA lysis buffer (Beyotime Biotechnology, Wuhan, Hubei, China) supplemented with a proteinase inhibitor cocktail (Roche, Basel, Switzerland). Cell lysates were centrifuged at 12,000 rpm for 10 min at 4°C, and the supernatant was harvested. The supernatant was frozen at -80°C for western blot analysis of protein expression.

Cytoplasmic and nuclei proteins were extracted using a cytoplasmic and nuclear protein extraction kit (Wuhan Boster Biotech) and subjected by western blotting. The blot was stripped and probed with anti-β-actin (for cytoplasmic extracts) or anti-Lamin B (for nuclear extracts) antibodies.

### Western Blotting

Extracted proteins were separated by SDS-PAGE on 10–15% gels, and then transferred onto a nitrocellulose membrane. Non-specific binding sites were blocked by 5% dried fat-free milk in Tris-buffered saline (TBS-T: 10 mmol/l Tris, 0.15 mol/l NaCl, 0.05% Tween-20, pH of the solution adjusted to 7.5). Primary antibodies were added and incubated at 4°C overnight as described previously. Membranes were washed with TBS-T, and then incubated with the secondary antibody, either goat anti-mouse IgG or anti-rabbit IgG horseradish peroxidase-conjugated antibody (1:5000). Bands of immunoreactive proteins were visualized on an image system (Versadoc; Bio-Rad) after membrane incubation with enhanced chemifluorescence (ECF) reagent for 5 min as described previously.

### Measurement of Reactive Oxygen Species (ROS)

Reactive oxygen species were measured using the non-fluorescent probe 2′7′-dichlorofluorescein diacetate (DCFH-DA, Beyotime, China). DCFH-DA passively diffuses into primary neurons and is deacetylated by esterases to form non-fluorescent 2′7′-dichlorofluorescein (DCFH). In the presence of ROS, DCFH reacts with ROS to form the fluorescent product DCF, which is trapped inside cells. Briefly, the culture medium was first removed and the cells were washed with PBS before incubation in DCFH-DA, diluted to a final concentration of 10 μM, for 20 min at 37°C. ROS levels were assessed by measuring the fluorescence at an excitation of 485 nm and an emission of 528 nm (Yang et al., 2017).

### Cell Viability Assays

Cell viability was evaluated using a CCK-8 assay kit (Beyotime Biotechnology, Hubei, China). Briefly, after each treatment, 10 mm of WST-8 reagent was added and primary neurons were incubated for 2 h at 37°C and 5% CO_2_. The absorbance of the samples was measured at 450 nm using a microplate reader with a background control as the blank. The cell survival ratio was expressed as the percentage of the control group (Song et al., 2014).

### Mitochondria Distribution and Morphology

To analyze mitochondrial distribution, treated-PCCN cells were incubated with pre-warmed (37°C) staining solution containing the Mitotracker Green FM probe (M7514) (Invitrogen) (final concentration 100 nM) for 45 min. After staining is complete, the staining solution was replaced with prewarmed fresh media and observed using an upright fluorescence confocal microscope (Olympus, Tokyo, Japan). The number of cells with non-tubular/fragmented mitochondria was determined, with at least 500 transfected cells examined. All quantifications were based on at least three independent experiments.

### Transmission Electron Microscopy (TEM)

Primary neurons were treated and then washed twice in PBS, trypsinized, fixed in ice-cold 5% glutaraldehyde in 0.1 M sodium cacodylate buffer (pH 7.4) at 4°C for 15 min, and then centrifuged. Cell pellets were fixed for 4 h. After a complete rinse with sodium cacodylate buffer, the cell pellet was further fixed in 1% OsO_4_ in 0.1 M sodium cacodylate buffer on ice for 1 h and dehydrated with acetone. The cell pellet was embedded in EM-bed 812 resin and polymerized at 60°C for 48 h. Ultrathin sections (70 nm) were obtained on a Leica Ultracut UCT ultramicrotome (Vienna, Austria) and counterstained with uranyl acetate and lead citrate before observation under a JEM-2100 TEM (JEOL, Japan).

### Statistical Analysis

All assays were performed on three separate occasions. Data were expressed as means ± SD. We have checked the distribution of all datasets and all were parametric. All comparisons for parametric data were made using Student’s test or one-way ANOVA followed by *post hoc* Turkey’s test using the SPSS software (version 13.0: SPSS Inc., Chicago, IL, USA), GraphPad Prism 5 software (La Jolla, CA, USA) and Image J software (National Institutes of Health, USA). *P* < 0.05 was considered statistically significant.

## Results

### REST is Downregulated in the Brain of Scrapie-Infected Experimental Animals

**FIGURE 1:**
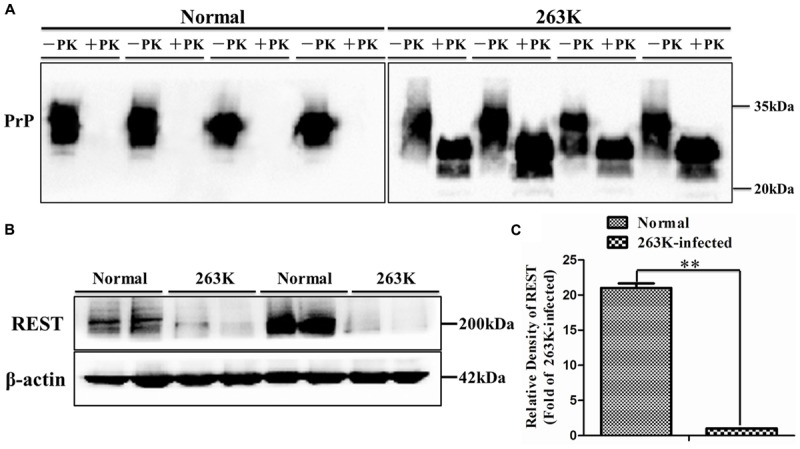
**Immunoblotting analyses of PrP^Sc^ and repressor element 1-silencing transcription (REST) in brain tissues of normal control and scrapie 263K-infected hamsters. (A)** Western blot analysis of PK-resistant PrP. **(B,C)** Immunoblotting density of REST was normalized to β-actin and values are expressed as fold changes relative to the 263K-infected hamsters. Data are presented as mean ± SD, *n* = 10. ^∗∗^*P* < 0.01 vs. the normal control. Statistical significance was evaluated using Student’s *t*-test.

**FIGURE 2:**
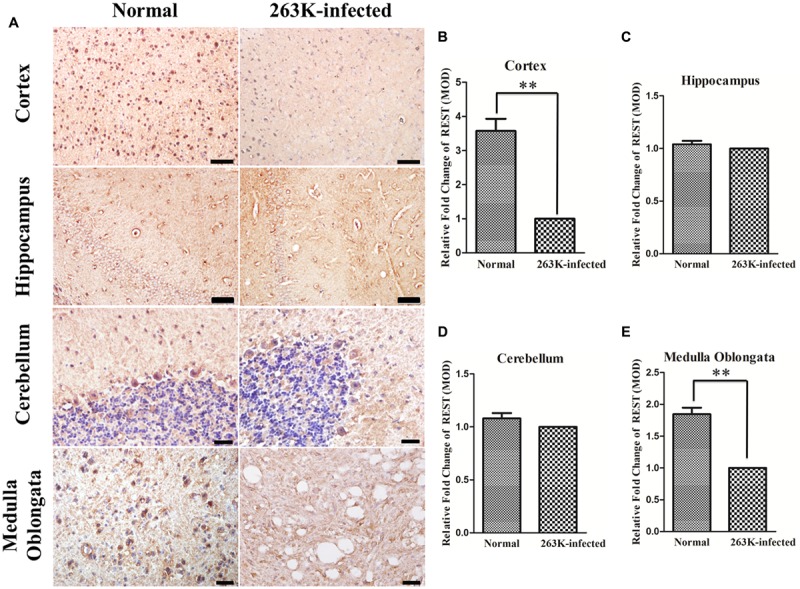
**Downregulation of REST expression in the brain of 263K-infected hamsters compared with normal controls. (A)** Immunohistochemical analysis of REST in the cortex and hippocampus (Scale bar = 50 μm), cerebellum and medulla oblongata (Scale bar = 20 μm) (DAB, yellow brown or dark brown). Nuclei were counterstained with haematoxylin (blue). **(B–E)** Mean optical density (MOD) of REST staining. Values are expressed as fold changes relative to the 263K-infected hamsters. All data are presented as mean ± SD, *n* = 10. ^∗∗^*P* < 0.01 vs. the normal control.

**FIGURE 3:**
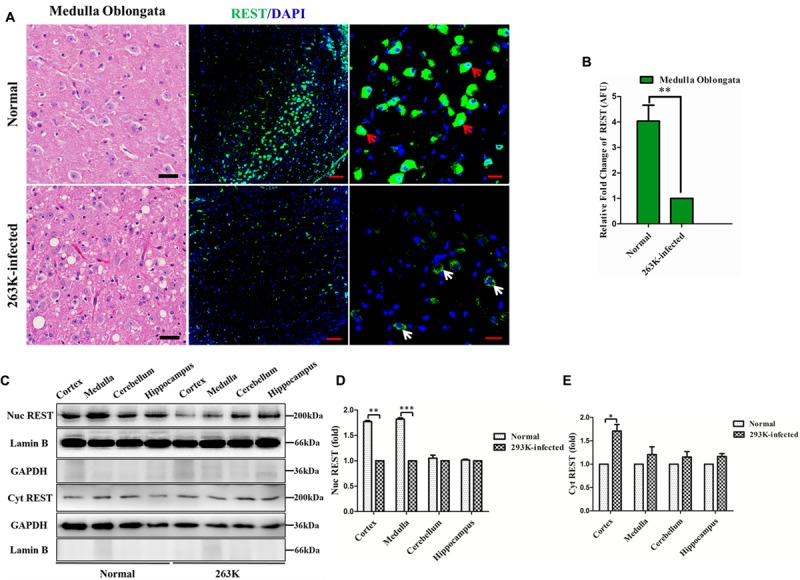
**Loss of REST from the nucleus in the brains of 263K-infected hamsters. (A)** Left panel: haematoxylin and eosin (H&E) staining showing the most severe lesions (vacuolation) in the medulla oblongata of 263K-infected hamsters. Scale bar = 20 μm. Middle panel: confocal immunofluorescence labeling for REST (green) and nucleus (DAPI, blue) in the medulla oblongata showing significantly decreased REST expression in 263K-infected hamsters relative to the normal control. Scale bar = 100 μm. Right panel: larger magnification of confocal photomicrographs of the middle panel showing the localization of REST. Red arrows show intense REST nuclear and cytoplasmic staining in the normal control; white arrows show typical cytoplasmic distribution of REST in the 263K-infected hamsters. Scale bar = 20 μm. **(B)** Quantitative analysis of REST levels in **(A)**. Relative arbitrary fluorescence units (AFU) values are expressed as fold changes relative to the 263K-infected hamsters. Data are presented as mean ± SD of triplicate experiments. ^∗∗^*P* < 0.01 vs. the normal control. **(C)** Immunoblotting of REST in the cytoplasmic and nuclear fraction of isolated cortex, medulla oblongata, cerebellum, and hippocampus of normal control and 263K-infected hamsters, respectively. GAPDH and the nucleus-localized protein Lamin B demonstrate separation of cytoplasmic and nuclear fractions. **(D,E)** Quantitative analysis of REST level (normalized to GAPDH or Lamin B) in the nucleus and cytoplasm in **(C)**, shown as the relative density to the 263K-infected hamsters. Data are presented as mean ± SD of triplicate experiments. ^∗^*P* < 0.05, ^∗∗^*P* < 0.01, ^∗∗∗^*P* < 0.001 vs. the normal control.

To investigate the expression of REST protein in scrapie-infected hamsters, we infected hamsters by intraperitoneal inoculation of 263 K prions and examined the amount of PK-resistant PrP in the brains of terminally sick hamsters (**Figure 1A**) firstly (Chen et al., 2014; Shah et al., 2017). The levels of REST in collected brain tissues were further analyzed by western blotting (**Figures 1B,C**). In line with a previous *in vitro* study (Song et al., 2016), REST markedly declined in the brain of 263K-infected hamsters at the terminal stage compared with the normal control. Secondly, cortex, cerebellum, hippocampus and medulla oblongata were immunohistochemically (IHC) studied for the expression of REST (**Figure 2**) and stained with haematoxylin and eosin (H&E) (**Figure 3A**, left panel) for observing and confirming brain lesions induced by 263K scrapie (Shah et al., 2017). Validation of the REST antibody specificity was carried out in a previous study (Song et al., 2016). Our analyses showed that REST was significantly downregulated in the cortex and medulla oblongata, associated with the most serious lesions in these regions of the brain, but not in the cerebellum or hippocampus (devoid of plaques or vacuoles) (**Figures 2B–E**) (**Figure 3**, introduced in the next result). In the control animals, the pattern of REST immunoreactivity was evenly distributed in both the cytoplasm and nucleus of all regions of the brains (**Figure 2A**, left panel) (**Figures 3C–E**). However, low expression of REST was observed in the cortex and medulla oblongata of 263K-infected hamsters, especially in the nucleus (**Figures 2A**, right panel, **B**) (**Figures 3C,D**). The marked down-regulation of REST in the 263K-infected hamsters in *in vivo* and *in vitro* experiments (Song et al., 2016) suggest that REST might be down-regulated in prion associated diseases and imply that REST might play a potential functional role in normal condition.

### REST is Lost from the Nucleus and Appears to Co-localize with LC3-II, a Marker of Cellular Autophagosomes, in the Cytoplasm of Neurons in 263K-Infected Hamsters

**FIGURE 4:**
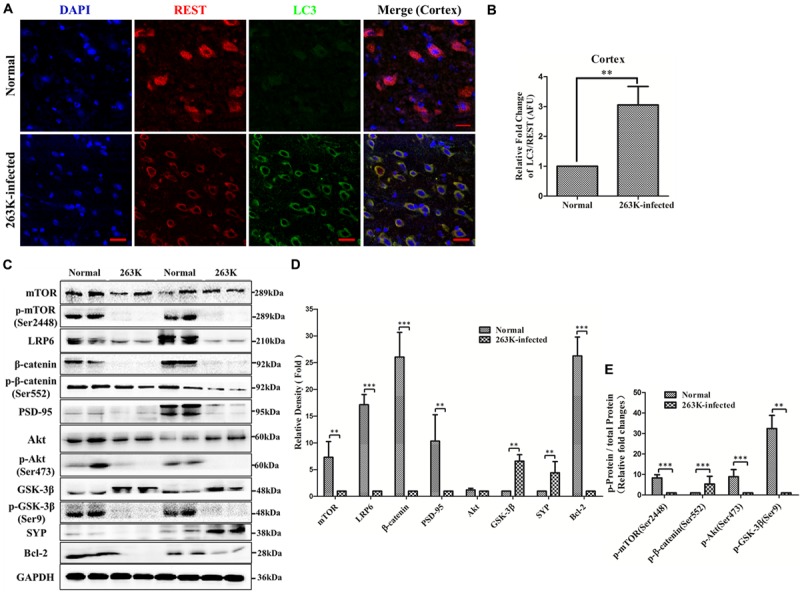
**(A,B)** Co-localization of REST with LC3-II, a marker of cellular autophagosomes, in the cytoplasm of the cortex in 263K-infected hamsters. **(A)** Confocal immunofluorescence labeling for REST (red), LC3-II (green) and nucleus (DAPI, blue) in the brain of the normal control and the 263K-infected hamsters. **(B)** Quantitative analysis of immunofluorescence in **(A)**. Relative AFU values (LC3-II/REST ratio) are expressed as fold change relative to the normal control. Data are presented as mean ± SD of triplicate experiments. ^∗∗^*P* < 0.01 vs. the normal control. **(C,D)** Analyses of Akt-mTOR and LRP6-Wnt-β-catenin signaling pathway molecules in brains of normal control and 263K-infected hamsters. **(C)** Immunoblots of total mTOR protein, p-mTOR (Ser2448), LRP6, total β-catenin protein, p-β-catenin (Ser552), PSD-95, total Akt protein, p-Akt (Ser473), total GSK-3β protein, p-GSK-3β(Ser9), Synaptophysin (SYP) and Bcl-2 in brain homogenates of normal and 263K-inefcted hamsters. **(D)** and **(E)** Quantitative analyses of **(C)**. Immunoblot density in **(D)** was normalized to GAPDH and expressed as the ratio to the GAPDH density. Immunoblot density in **(E)** showed the quantification of p-mTOR (Ser2448)/total mTOR protein, p-β-catenin (Ser552)/total β-catenin protein, p-Akt (Ser473)/total Akt protein and p-GSK3β (Ser9)/total GSK3β protein, total mTOR, β-catenin, Akt and GSK3β protein were normalized to GAPDH. Data are presented as mean ± SD of triplicate experiments. Immunoblotting density was expressed as a ratio to the less amount group for each protein, ^∗∗^*P* < 0.01, ^∗∗∗^*P* < 0.001 vs. the normal control.

To obtain further information on the alteration of REST, the distribution of REST was directly observed by immunofluorescence in the medulla oblongata (**Figures 3A,B**) or cortex (**Figure 4A**, the second row)slices of scrapie-infected hamsters since those regions showed a significant alteration of REST in the previous data. Additionally, subcellular localization of REST in the nucleus and cytoplasm fractions of isolated cortex, medulla oblongata, cerebellum, and hippocampus of normal control and 263K-infected hamsters were quantified by western blotting (**Figure 3C**) using GAPDH and Lamin B as the cytoplasmic and nuclear marker, respectively (**Figures 3D,E**). As expected, in agreement with the IHC results, REST was sparsely distributed in the nucleus of cortex and medulla in the 263K-infected hamsters compared to the normal control (**Figure 3D**). Cytoplasmic levels of REST were low and comparable between the infected and control groups (exclude cortex) (**Figure 3E**). The relatively increased level of REST in the cortex of 263K-infected hamsters compared with the normal control in line with our previous *in vitro* experiments (Song et al., 2016), suggesting the translocation of REST from nucleus to cytoplasm in prion diseases. In summary, these data highlight a speculation that there is a linkage between the loss of REST from the nucleus and its dysfunction in 263K-infected hamsters.

Furthermore, previously, autophagic vacuolation and hyperactivation of the autophagic system in neurons of prion diseases were observed under electron microscope (Boellaard et al., 1991; Jeffrey et al., 1995). We previously found that overexpression of REST alleviated PrP106-126-induced excess autophagosomes or autophagolysosomes in PCCN (Song et al., 2016). To examine the relationship of REST and autophagy in the cortex, brain sections of normal and 263K-infected hamsters were double-stained with antibodies to REST and LC3-II (a marker of cellular autophagosomes). In 263K-infected hamsters, loss of REST from the nucleus was obviously accompanied by accumulation of autophagosomes in cytoplasm (**Figures 4A,B**) consistent with the data shown in **Figures 3C–E**.

### Inactivation of the Akt-mTOR and Partial LRP6-Wnt-β-Catenin Signaling Pathways in 263K-Infected Hamsters

The Akt-mTOR pathway is an important negative signal for autophagy in mammalian cells (Shimobayashi and Hall, 2014; Xu et al., 2014). The macroautophagic system in neurons is activated partially through the mTOR pathway in prion diseases (Xu et al., 2014) and Alzheimer’s disease (Lafay-Chebassier et al., 2005). Autophagy of REST was also observed in age-related neurodegenerative disorders (Lu et al., 2014). Additionally, we previously reported that the expression of REST was induced by the LRP6-Wnt-β-catenin signaling pathway in PCCN (Song et al., 2016). We postulated that the Akt-mTOR signaling pathway might be involved in REST suppression in prion diseases. Therefore, total mTOR protein, p-mTOR (Ser2448), total Akt protein, p-Akt (Ser473), LRP6, total β-catenin protein, p-β-catenin (Ser552), total GSK3β protein, and p-GSK3β(Ser9), which are hallmarks of the Akt-mTOR and LRP6-Wnt-β-catenin pathways, in brain tissues of the normal and 263K-infected hamsters were analyzed by western blotting. The expression of total mTOR protein and p-mTOR (Ser2448)/total mTOR protein were reduced to various extents (**Figures 4C–E**). In particular, p-Akt (Ser473) and p-mTOR (Ser2448), biochemical indicators of Akt and mTOR activation (Hay and Sonenberg, 2004), were decreased to an almost undetectable level in the brains of scrapie-infected hamsters, suggesting nearly complete inactivation of the Akt-mTOR pathway, whereas levels of total Akt protein had no significant change. Relative expression of p-Akt (Ser473)/total Akt protein in the normal control was 5.18–12.25-folds than the 263K infected group (**Figures 4C,D**).

The essential co-receptor for Wnt signaling, the low-density lipoprotein receptor-related protein 6 (LRP6) (Niehrs, 2012), which showed a transient decrease after incubation with neurotoxic prion peptide in PCCN in our previous study (Song et al., 2016), was decreased more than 14-fold compared with the normal control. Notably, the glycosylated form of LRP6 (shown as the lower-mobility/upper band in the LRP6 line of **Figure 4C**) were observed in the normal group but disappeared in the 263K infected group. This *in vivo* experiment indirectly confirms our previous data that LRP6-mediated Wnt-β-catenin signaling partly regulates the expression of REST (Song et al., 2016). β-catenin (Zhang et al., 1998) and GSK3β (Perez et al., 2003), as the indicator of activation and inhibition of the Wnt-β-catenin pathway, respectively, were also examined. Both total and the phosphorylated form were analyzed because phosphorylation at Ser552 induces β-catenin accumulation in the nucleus and increases its transcriptional activity (He et al., 2007), while the phosphorylation of GSK3β at Ser9 suggesting an inhibitory state (Cross et al., 1995). Interestingly, the levels of total β-catenin in the 263K infected group were significantly down-regulated, while p-β-catenin (Ser552)/total β-catenin protein were increased compared with the normal control. p-GSK3β (Ser9)/total GSK3β protein were significantly decreased, while total GSK3β was increased compared with the normal control (**Figures 4C,D**). β-catenin is a direct substrate of GSK-3β and an intermediate effector of the canonical Wnt signaling pathway (Yost et al., 1996; Singh et al., 2016). GSK-3 activity is also regulated by a wide variety of kinases and systems including the Wnt pathway, Akt, protein kinase A (PKA), protein kinase C (PKC), and MAP kinases (Cross et al., 1995; Chiu and Chuang, 2010). The data showed a result mediated by a complicated network, thereby providing for a regulation of different outcomes. These present studies demonstrated the inhibition of LRP6-Wnt-β-catenin signaling in 263K-infected hamsters. Furthermore, consistent with the previous data in PCCN (Song et al., 2016), a significant decline of the postsynaptic protein marker, PSD-95, was observed, confirming synaptic damage. The antiapoptotic protein Bcl-2 (B cell lymphoma-2) markedly decreased, demonstrating the alteration of apoptotic signaling. Thus, both of the Akt-mTOR and part of LRP6-Wnt-β-catenin signaling pathways were inhibited in 263K-infected hamsters, which might have contributed to the downregulation of REST.

### Suppression of the Akt-mTOR and LRP6-Wnt-β-Catenin Signaling Pathways in PCCN by the Neurotoxic Prion Peptide, PrP106-126

**FIGURE 5:**
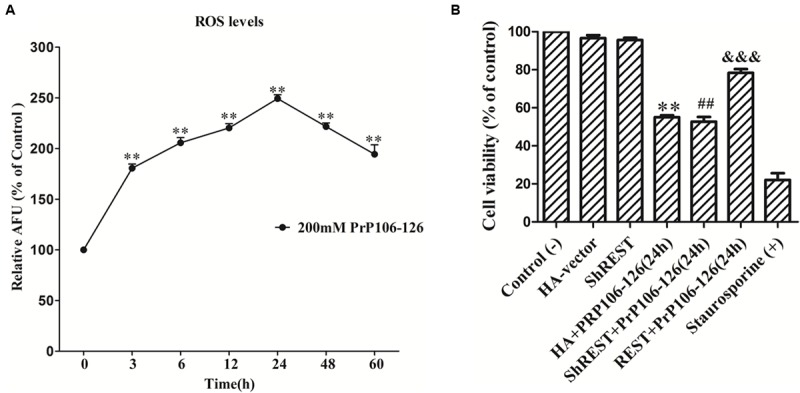
**PrP106-126-induced neurotoxicty in primary cultured cortical neurons (PCCN). (A)** PrP106-126 induced the increase in intracellular reactive oxygen species (ROS) levels. PCCN were treated with PrP106–126 (200 μM) for various time points (3–60 h). ROS levels were determined by the ROS probe, DCFH-DA. Data are presented as mean ± SD in triplicate experiments. ^∗∗^*P* < 0.01 vs. the normal control (0 h). **(B)** Protective effects of REST in cell viability measured by the CCK-8 assay. PCCN were transfected with the HA vector or shREST vector or REST-HA vector and incubated with or without PrP106-126 for 24 h. Staurosporine = positive control (+). The values are percent viable cells relative to the control (–) (no treatment). The data were analyzed using a one-way ANOVA followed by *post hoc* Tukey’s multiple comparison tests. ^∗∗^*P* < 0.01, ^##^*P* < 0.01 vs. the control (–). ^&&&^*P* < 0.01 are ShREST+PrP106-126, PrP106-126+HA-vector vs. the PrP106-126+REST-HA Vector group, respectively. HA, HA-vector.

As a widely used model for the *in vitro* study of prion diseases, neurotoxic prion peptide (PrP106-126) is used as a material in our further research *in vitro* in PCCN. PrP106-126-induced neurotoxicity and pathological damage in PCCN had been proved in our previous studies (Song et al., 2014, 2016; Zhu et al., 2015; Yang et al., 2017). Here, we confirmed the neurotoxicity of PrP106-126 by more approaches. A time course analysis of ROS levels following PrP106-126 (200 μM) stimulation in PCCN was performed, utilizing the 2′,7′- dichlorodihydrofluorescein fluorescent probe. **Figure 5A** showed PrP106-126-induced oxidative stress (*P* < 0.01) with prolonged response time. Cell viability in different treated group was detected using the CCK-8 assay and treatment with staurosporine (500 nm for 24 h) (Bertrand et al., 1994; Song et al., 2016) as a positive control of neuronal death. The data demonstrated: (1) Compared with the results in the control group, the number of cell viability in the PrP106-126-treated group was decreased by 45.0% (*P* < 0.01); (2). Consistent with our previous data (Song et al., 2016), after the stimulation of PrP106-126, compared with the HA-vector (55.0% viability) (*P* < 0.01) and shREST (52.6% viability) (*P* < 0.01) groups, REST-overexpression significantly increased cell viability (78.3% viability) (*P* < 0.001) (**Figure 5B**).

**FIGURE 6:**
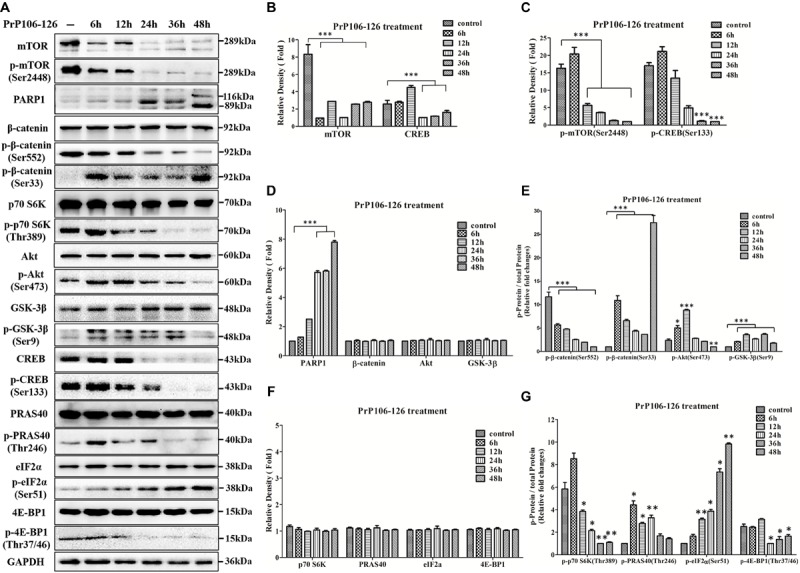
**Suppression of the Akt-mTOR and LRP6-Wnt-β-catenin signaling pathways in PrP106-126-stimulated primary cultured cortical neurons (PCCN). (A)** Immunoblotting of total mTOR protein, p-mTOR (Ser2448), PARP1, total β-catenin protein, p-β-catenin (Ser552), p-β-catenin (Ser33), p-p70 S6K (Thr389), total Akt protein, p-Akt (Ser473), total GSK-3β protein, p-GSK-3β (Ser9), total CREB protein, p-CREB (Ser133), total PRAS40 protein, p-PRAS40 (Thr246), total eIF2α protein, p-eIF2α (Ser51), total 4E-BP1 protein and p-4E-BP1 (Thr37/46) in PCCN exposed to PrP106-126 at various times (6–48 h). **(B–F)** Quantitative analyses of **(A)**. Immunoblot density in **(B,D,F)** were normalized to GAPDH and expressed as ratio to the minimum expression (at various times during incubation). Immunoblot density in **(C)** showed the quantification of p-mTOR (Ser2448)/total mTOR and p-CREB (Ser133)/total CREB; **(E)** showed the quantification of p-β-catenin (Ser552)/total β-catenin, p-β-catenin (Ser33)/total β-catenin, p-Akt (Ser473)/total Akt protein and p-GSK3β (Ser9)/total GSK3β protein; **(G)** showed the quantification of p-p70 S6K (Thr389)/total p70 S6K, p-PRAS40 (Thr246)/total PRAS40, p-eIF2α (Ser51)/total eIF2α and p-4E-BP1 (Thr37/46)/total 4E-BP1. All the total protein were normalized to GAPDH. Data are presented as mean ± SD of triplicate experiments. ^∗^*P* < 0.05, ^∗∗^*P* < 0.01, ^∗∗∗^*P* < 0.001 vs. PBS control group (–).

Next, to further explore the potential relationship between REST and dysfunctional regulatory pathways, we also determined if the Akt-mTOR and LRP6-Wnt-β-catenin signaling pathways were altered in PCCN after exposure to PrP106-126. PCCNs were treated with 200 μM PrP106-126, and the protein extract at various time points (0–48 h) was analyzed by western blotting. Consistent with the *in vivo* data in infected hamsters, total mTOR protein and p-mTOR (Ser2448)/total mTOR protein declined with prolonged stimulation by the prion peptide compared with the PBS control group (*P* < 0.001) (**Figures 6A–C**); Although levels of total Akt protein had no significant change (**Figures 6A,D**), p-Akt (Ser473)/total Akt protein showed a transient increase after incubation for 6–12 h and then declined with prolonged stimulation compared with PBS control group (**Figures 6A,E**). Furthermore, total and phosphorylated forms of proteins downstream of Akt-mTOR, including total PRAS40 protein, p-PRAS40 (Thr246), total p70 S6K protein, p-p70 S6K (Thr389), total 4EB-P1 protein and p-4E-BP1 (Thr37/46), were examined. All the total forms of these proteins had no significant change (**Figures 6A,F**). Notably, the phosphorylated forms of these proteins (normalized to corresponding total protein) had a tendency to decrease, demonstrating strong inhibition of the Akt-mTOR pathway by the neurotoxic prion peptide (**Figures 6A,G**).

On the other hand, although total β-catenin and GSK3β had no significant changes after treatment with PrP106-126 (**Figures 6A,D**) in line with our previous *in vitro* studies (Song et al., 2016), p-β-catenin (Ser552)/total β-catenin protein declined time-dependently in PCCN incubated with PrP106-126 (*P* < 0.001) (**Figures 6A,E**). The different outcomes of normalized p-β-catenin (Ser552) *in vivo* and *in vitro* might due to the different treated time in each prion diseases models and varying response of molecules to the course of diseases. p-β-catenin (Ser33)/total β-catenin protein was significantly induced by the peptide (*P* < 0.001) (**Figures 6A,E**), indicating β-catenin destabilization (Yost et al., 1996). Phosphorylation of GSK3β at Ser 9 is involved in the inactivation of GSK3 (Cohen and Frame, 2001; Perez et al., 2003). p-GSK3β (Ser9)/total GSK3β protein was almost undetectable in the PBS control group, but it became activated for degeneration of GSK3β to restore normal level in PCCN. However, the ability for reducing the production of GSK3β was weaker with prolonged stimulation by PrP106-126 (*P* < 0.001) (**Figures 6A,E**). The above data demonstrated the partially inactivation of LRP6-mediated Wnt-β-catenin signaling pathway in prion peptide-treated neuronal cells, consistent with the *in vivo* findings in 263K-infected hamsters and results of our previous study (Song et al., 2016).

We previously showed that PrP106-126 induced caspase-3 activation. Here, Poly (ADP-ribose) Polymerase (PARP), one of the main cleavage targets of caspase-3, was examined. PARP maintains cell viability, and cleavage of PARP facilitates cellular disassembly and serves as a marker of apoptosis (Tewari et al., 1995). PARP in PCCN was cleaved to an 89 kDa apoptotic fragment after exposure to PrP106-126 (**Figures 6A,D**). Total and phosphorylated forms of CRE-binding protein (CREB) were tested as CREB plays a key role in promoting neuronal survival and axon extension (Lonze et al., 2002). Total and Phosphorylation of the eukaryotic initiation factor 2 (eIF2) α subunit (Sheikh and Fornace, 1999) was also analyzed to evaluate the stress condition. As expected, both CREB forms were markedly suppressed (**Figures 6A–C**), level of total eIF2α protein had no significant change (**Figures 6A,D**) while p-eIF2α (Ser51)/total eIF2α protein was significantly induced by PrP106-126 over time (**Figures 6A,F,G**).

In summary, the neurotoxic prion peptide, PrP106-126 inhibited the Akt-mTOR and Wnt-β-catenin signaling pathways, associated with the downregulation of REST, and disrupted the neuronal survival pathway by promoting apoptosis and stress in PCCN.

### Overexpression of REST Alleviates PrP106-126-Induced Inactivation of the Akt-mTOR and Wnt-β-Catenin Pathways

**FIGURE 7:**
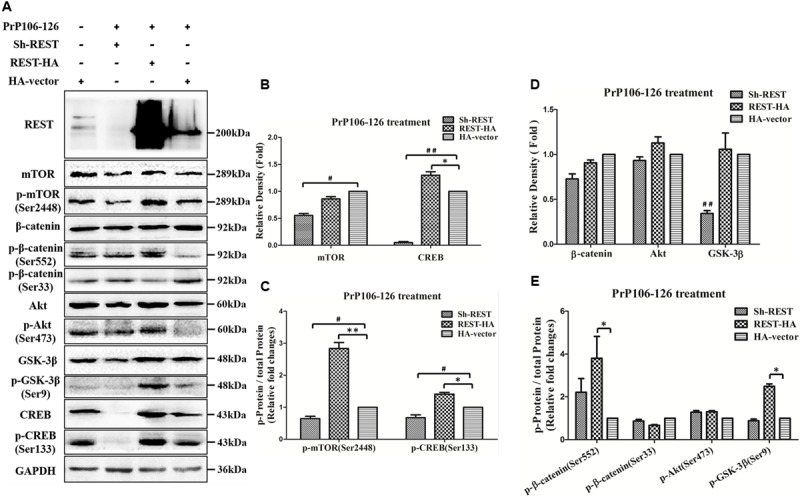
**Overexpression of REST restores normal levels of phosphorylated Akt-mTOR and Wnt-β-catenin associated hallmarks and p-CREB (Ser133) protein in PrP106-126-stimulated PCCN. (A)** PCCNs were transfected with a control HA-vector, or REST shRNA (REST knockdown) or REST-vector (REST overexpression) and incubated with PrP106-126. Cellular proteins were immunoblotted for total mTOR protein, p-mTOR (Ser2448), total β-catenin protein, p-β-catenin (Ser552), p-β-catenin (Ser33), total Akt protein, p-Akt (Ser473), total GSK-3β protein, p-GSK-3β (Ser9), total CREB protein and p-CREB (Ser133). **(B–E)** Quantitative analyses of **(A)**. Immunoblotting density in **(B)** and **(D)** were normalized to GAPDH and expressed as relative density to the HA-vector (PrP106-126 treated) positive control group (HA-vector transfected and treated with PrP106-126). Immunoblot density in **(C)** showed the quantification of p-mTOR (Ser2448)/total mTOR and p-CREB (Ser133)/total CREB, **(E)** showed the quantification of p-β-catenin (Ser552)/total β-catenin, p-β-catenin (Ser33)/total β-catenin, p-Akt (Ser473)/total Akt protein and p-GSK3β (Ser9)/total GSK3β protein. All the total protein were normalized to GAPDH. Data are presented as mean ± SD of triplicate experiments. ^∗^*P* < 0.05, ^∗∗^*P* < 0.01 is REST-HA (PrP106-126 treated) group vs. HA-vector (PrP106-126 treated) group, ^#^*P* < 0.05, ^##^*P* < 0.01 is sh-REST (PrP106-126 treated) group vs. HA-vector (PrP106-126 treated) group.

We overexpressed or blocked REST as described before (Song et al., 2016) and then treated PCCN with 200 μM PrP106-126. A HA vector-transfected and untreated and a HA vector-transfected and treated group served as the negative and transfection controls, respectively. REST protein levels were determined by Western blotting. We determined Ser-473-phosphoralated and total forms of Akt and Ser-2448-phosphoralated and total forms of mTOR to see whether REST expression affects the Akt-mTOR pathway (**Figures 7A–C**). Examined total and phosphorylation of β-catenin and GSK3β to evaluate the effect of REST on the Wnt-β-catenin pathway (**Figures 7A,D,E**). The results demonstrated that, under the stimulation of PrP106-126, overexpression of REST: **(1)** enhanced p-mTOR (Ser2448)/total mTOR protein (by 2.84-fold relative to the HA-vector group), p-β-catenin (Ser552)/total β-catenin protein (by 3.80-fold relative to the HA-vector group) and p-GSK3β (Ser9)/total GSK3β protein (by 2.49-fold relative to the HA-vector group); **(2)** alleviated PrP106-126-induced decrease of both total form and phosphorylation (Ser133) form (normalized to total protein)of CREB by 1.30- and 1.41-fold, relative to the HA-vector group, respectively. (3) overexpression of REST significant restored the levels of mTOR, p-mTOR (Ser2448), p-β-catenin (Ser552), total and phosphorylation (Ser9) forms of GSK3β compared with the knockdown of REST group. Conversely, under the PrP106-126-induced pathological conditions, knockdown of REST exacerbated the inactivation of these proteins (**Figure 7**) compared with the HA-vector group, further suggesting the important and essential role of REST in the regulation of Akt-mTOR and Wnt-β-catenin signaling pathways in neuronal cells.

**FIGURE 8:**
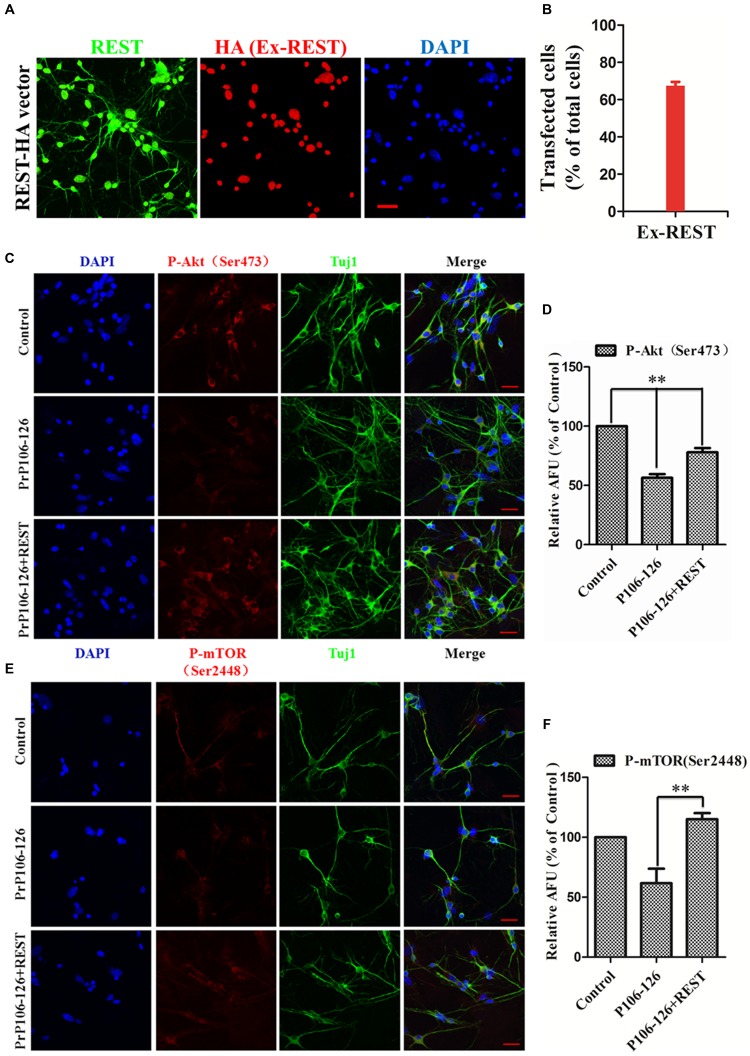
**Overexpression of REST alleviates PrP106-126-induced suppression of Akt phosphorylation at Ser473 and mTOR phosphorylation at Ser2448. (A)** Confocal immunofluorescence labeling for total REST (green), exogenously expressed (ex-REST) REST (Red) and nucleus (DAPI, blue). Exogenous REST was stained with HA antibody (Red). **(B)** Quantitative analysis of **(A)** to show the transfection efficiency of PCCN. **(C,E)** Confocal immunofluorescence images of p-Akt (Ser473) (red) and p-mTOR (Ser2448) (red) in PCCN transfected with HA-vector or REST-vector, with or without incubation with PrP106-126. Tuj1 (green) is used as a neuronal marker. Scale bar = 30 μm. **(D,F)** Quantitative analysis of immunofluorescence in **(C,E)**, respectively. Relative AFU values are expressed as percentage of the control. Data are presented as mean ± SD of triplicate experiments. ^∗∗^*P* < 0.01 vs. PrP106-126-treated group.

**FIGURE 9:**
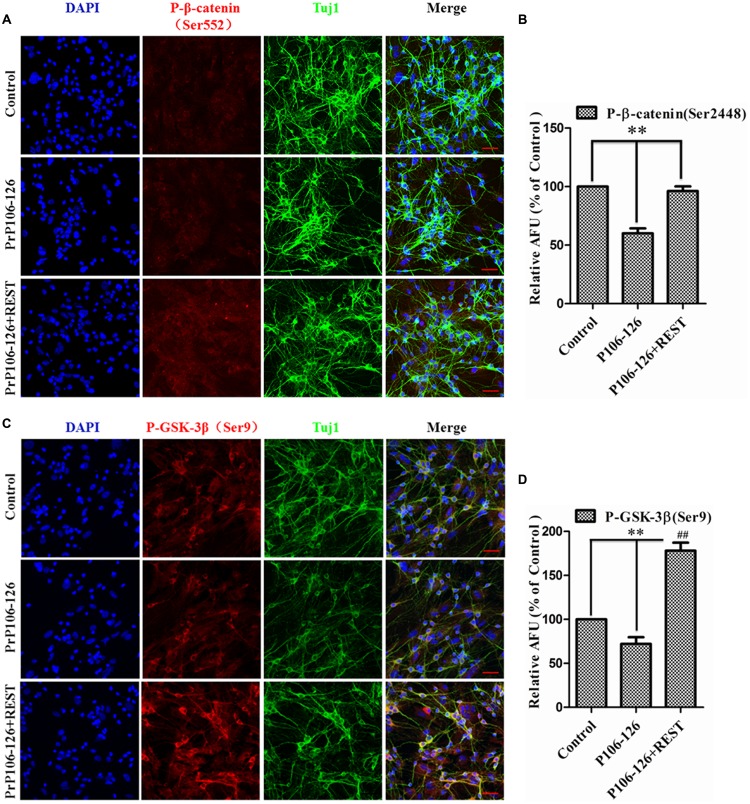
**Overexpression of REST alleviates PrP106-126-induced suppression of β-catenin phosphorylation at Ser552 and GSK-3β at Ser9. (A,C)** Confocal immunofluorescence images of p-β-catenin (Ser552) (red) and p-GSK-3β (Ser9) (red) in PCCN transfected with HA-vector or REST-vector with or without incubation with PrP106-126. Tuj1 (green) was used as a neuronal marker. Scale bar = 40 μm. Scale bar = 10 μm. **(B,D)** Quantitative analysis of immunofluorescence in **(A,C)**, respectively. Relative AFU values are expressed as percentage of the control. Data are presented as mean ± SD of triplicate experiments. ^∗∗^*P* < 0.01 vs. PrP106-126-treated group.^##^*P* < 0.01 is REST-HA (PrP106-126 treated) group vs. control group (HA-vector transfected and untreated).

To further directly observe and confirm the correlation of REST expression and Akt–mTOR and Wnt-β-catenin pathways in the prion disease model, p-Akt (Ser473), p-mTOR (Ser2448), p-β-catenin (Ser522), and p-GSK3β (Ser9) levels were quantified in PrP106-126-treated PCCN by immunofluorescence and expressed as percent of the HA-vector-transfected, untreated negative control. Firstly, confocal immunofluorescence labeling for total REST (green), exogenously expressed REST (Red) (stained with HA antibody) and nucleus (DAPI, blue) was quantitative analysis to show the transfection efficiency of exogenous REST in PCCN (**Figures 8A,B**). Consistent with the western blotting results, REST significantly prevented the inhibition of the phosphorylation of Akt, mTOR (**Figures 8C–F**), β-catenin and GSK3β in PCCN by PrP106-126 (**Figures 9A–D**). Compared with the negative control (set as 100%), the relative arbitrary fluorescence units (AFU) of p-Akt (Ser473), p-β-catenin (Ser522) and p-GSK3β (Ser9) were markedly suppressed by 56, 62, 60, and 72%, respectively, by PrP106-126. Overexpression of REST recovered the levels of phosphorylated Akt (78% of the negative control), mTOR (115%), β-catenin (96%), GSK3β (178%), thereby alleviating PrP106-126-mediated inactivation of the Akt-mTOR and Wnt-β-catenin pathways.

### REST Acts as a Neuroprotective Regulator and Protects against PrP106-126-Induced Oxidative Stress and Mitochondrial Damage

**FIGURE 10:**
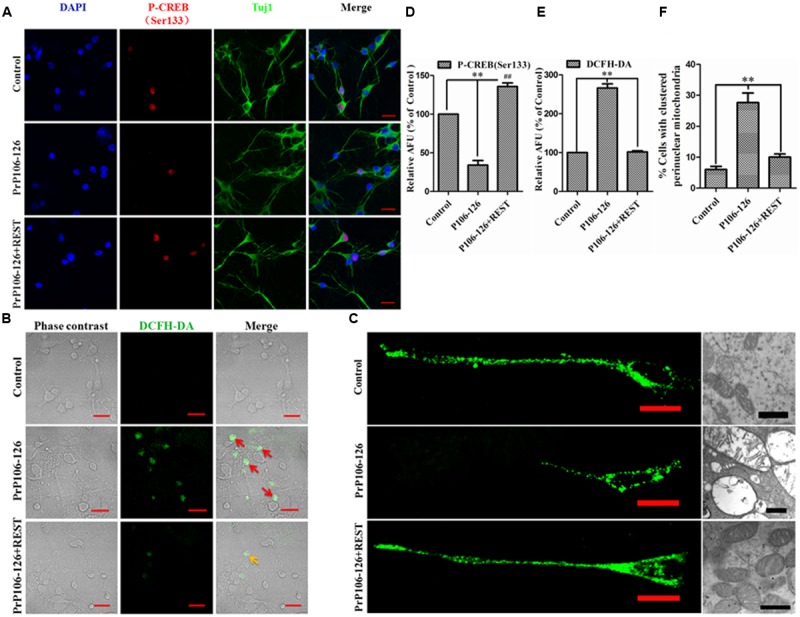
**Repressor element 1-silencing transcription acts as a neuroprotective regulator. (A)** Confocal immunofluorescence images of p-CREB (Ser133) (red) in PCCN transfected with HA-vector or REST-vector, with and without exposure to PrP106-126 as indicated. Tuj1 (green) is used as a neuronal marker. **(B,E)** REST protects against P106-126-induced oxidative stress. ROS production was visualized by fluorescence microscopy using the ROS probe, DCFH-DA. The relative AFU values are measured for at least 500 cells and are shown in the graph **(E)**. Data are presented as mean ± SD of triplicate experiments. ^∗∗^*P* < 0.01 vs. PrP106-126-treated group. **(D)** Quantitative analysis of immunofluorescence in **(A)**. Relative AFU values are expressed as percentage of the control. Data are presented as mean ± SD of triplicate experiments. ^∗∗^*P* < 0.01 vs. PrP106-126-treated group. ^##^*P* < 0.01 is REST-HA (PrP106-126 treated) group vs. control group (HA-vector transfected and untreated). **(C,F)** REST decreases the number of cells harboring perinuclearly clustered mitochondria stimulated by P106-126. PCCN were stained with MitoTracker Green to visualize mitochondria and analyzed by fluorescence microscopy. Right panel **(C)** shows the impairment of mitochondria by TEM. For quantification, mitochondria of at least 500 cells per experiment were determined in a blinded manner. Quantifications were based on triplicates of at least three independent experiments. ^∗∗^*P* < 0.01 vs. PrP106-126-treated group. Scale bar = 20 μm.

In consideration of the function of both CREB and REST in the synaptic plasticity and neuroprotection (Wu and Xie, 2006), increased CREB activation by GSK3β phosphorylation at Ser-9 (Grimes and Jope, 2001), and increased p-GSK3β (Ser9) by REST overexpression, the Ser-133-phosphorylated CREB was examined. Overexpression of REST markedly increased phosphorylation and activation of CREB, further demonstrating the function of REST in synaptic integrity and neuroprotection. Conversely, blocking REST considerably inhibited CREB phosphorylation to an almost undetectable level (**Figures 7A,C**). The location and expression of p-CREB (Ser133) was further observed by immunofluorescence. As expected, REST overexpression significantly increased p-CREB to 135% from 34.1% in the group without REST transfection (**Figures 10A,D**). As our previous reports showed that REST enhanced the expression of transcription factor FOXO1, an anti-apoptotic protein, which also mediates oxidative stress resistance (Kajihara et al., 2006; Tothova et al., 2007) and is protected by REST in AD (Lu et al., 2014), we further explored the correlation of REST with oxidative stress in our *in vitro* prion disease model. PrP106-126 treatment in PCCN significantly increased ROS production compared with the control culture, as revealed by 2′,7′-dichlorodihydrofluorescein (DCFH) fluorescent probe (green). ROS was suppressed by REST overexpression (**Figures 10B,E**).

Since mitochondria are critical regulators of cell survival and death in neurodegeneration (Lin and Beal, 2006), we assessed the distribution of mitochondria in primary neurons. Exposure of PCCN to PrP106-126 significantly increased the number of cells harboring clustered perinuclear mitochondria. However, transfection of neuronal cells with the REST vector before exposure to PrP106-126 markedly decreased mitochondrial changes induced by the prion peptide (**Figures 10C,F**). Combining with our previous data that overexpression of REST restored mitochondrial function and alleviated PrP106-126-induced abnormal mitochondrial structures (Song et al., 2016), we can summarize that REST works as a neuroprotective factor partially through protecting the function, integrity and normal distribution of mitochondria in PCCN.

## Discussion

### REST Is Depleted in Prion and Other Neurodegenerative Diseases

Nucleocytoplasmic transport is considered an important event for REST as a transcriptional repressor to regulate neuronal gene expression (Chu et al., 2007; Hung and Link, 2011; Patel and Chu, 2011). We previously reported that REST was induced and translocated from the cytoplasm to the nucleus in PCCN upon exposure to the prion peptide but failed to function as a neuroprotective factor with continued stimulation by PrP106-126 (Song et al., 2016). Here we further demonstrated that REST is lost from the nucleus in the brains of scrapie-infected hamsters and taken up in autophagosomes. Other recent studies also suggest that REST is induced in the aging brain but markedly depleted in the nucleus of neurons in Alzheimer’s disease, frontotemporal dementia and dementia with Lewy bodies (Lu et al., 2014). The phenomenon of protein shuttling between the nucleus and cytoplasm is also an important mechanism in regulating apoptosis and maintaining the basal activities of transcription factors by signaling molecules (Hung and Link, 2011; Patel and Chu, 2011). Taken together, the depletion of REST from nucleus may be a universal feature in prion and associated neurodegenerative diseases.

### REST Acts as a Mediator of the Akt-mTOR and Wnt-β-Catenin Pathways

Alteration of the autophagic system in neurodegenerative disorders including prion diseases is a widespread phenomenon (Parr et al., 2012; Xu et al., 2014). Consistent with this hypothesis, we previously observed excessive amounts of autophagosomes or autophagolysosomes in neurotoxic prion peptides-treated primary neurons, implying the activation of the autophagic system (Song et al., 2016). Here we further showed that Akt-mTOR signaling, the most important negative pathway for autophagy in mammalian cells is inhibited in prion diseases models *in vitro* and *in vivo*. REST co-localizes with the autophagosome marker, LC3-II in the cytoplasm in the brain of 263K-infected hamster but not in un-infected animals, suggesting that the activation of autophagic system is involved in the depletion of REST from the nucleus and contributes to the degradation of REST in the cytoplasm. Moreover, Wnt-β-catenin signaling, which partially induces the expression of REST under normal conditions, is suppressed in prion diseases. Alterations in the Akt-mTOR (Heras-Sandoval et al., 2014) and Wnt-β-catenin (Niehrs, 2012; Inestrosa and Varela-Nallar, 2014) pathways are both linked to the pathology of neurodegenerative diseases. A recent study demonstrated that knockdown of REST disrupted the mTOR signaling pathway and reduced cell viability in human oral SCC cells in a time-dependent manner, leading to cell apoptosis and DNA fragmentation (Cho et al., 2014).

We further investigated the role of REST in the Akt-mTOR and Wnt-β-catenin pathways in our *in vitro* prion disease model. We found that overexpression of REST in P106-126-treated primary neurons restores the normal levels of phosphorylated Akt-mTOR and Wnt-β-catenin proteins and represses the formation of autophagic vacuoles; whereas knockdown of REST exacerbates the inactivation of Akt-mTOR and Wnt-β-catenin pathways and the damage of organelle ultrastructures induced by the prion peptide PrP106-126. For the first time, our data suggest that REST plays an essential role in the regulation of both the Akt-mTOR and Wnt-β-catenin signaling pathways via phosphorylation of proteins involved in the pathways. Moreover, it has been proposed that the N-linked glycosylation of LRP6 in the endoplasmic reticulum (ER) is necessary for the maturation and cell surface location of this receptor (Khan et al., 2007; Abrami et al., 2008). Conversely, retention of LRP6 through blocking of its maturation may make cells insensitive to Wnt ligands, which specifically activate the Wnt/β-catenin pathway (Jung et al., 2011). In line with our previous study, the glycosylated form of LRP6 disappeared in the 263K infected group might be a potential reason to the downregulation of REST in prion disease. In summary, we propose a model to explain how REST-mediated neuroprotection is lost in prion diseases and further prove the important role of REST in mediating the upstream and downstream signaling pathways. This regulative signaling could be explored as a novel therapeutic target for prion diseases and associated neurodegenerative diseases.

### REST is an Essential Neuroprotective Regulator in Prion Diseases

Repressor element 1-silencing transcription plays a key role in neuron development. Perturbation of REST expression or function results in early embryonic lethality and ectopic expression of neuronal genes in non-neuronal tissues (Chen et al., 1998; Ballas et al., 2005). Lu et al. (2014) have shown that REST protein protects aging neurons from death by repressing genes that promote cell death and AD pathology and inducing the expression of stress response genes. REST and its functional pathways were found to be protective in hippocampal atrophy in amnestic mild cognitive impairment (Nho et al., 2015). By overexpressing or silencing REST in PCCN exposed to PrP106-126, our prior study identified that REST contributes to neuronal viability by stabilizing the pro-survival protein FOXO1 and the mitochondrial outer membrane and inhibiting the release of cytochrome c from mitochondria to cytosol and its downstream activation of Capase3 (Song et al., 2016). In the present study, we further showed that overexpression of REST not only restores normal levels of phosphorylated Akt-mTOR and Wnt-β-catenin proteins but also stabilizes the total and phosphorylated forms of CREB proteins, the latter of which is an important transcription factor regulating a wide-range of neuronal functions including neuronal survival, neuronal proliferation and differentiation, process growth, and synaptic plasticity (Lonze et al., 2002; Redmond et al., 2002). As comparative sequence analysis has revealed an intricate network among REST, CREB, and miRNA in mediating neuronal gene expression (Wu and Xie, 2006), their relationships deserve further research. Additionally, REST inhibits P106-126-induced ROS production and rescues the function, integrity and normal distribution of mitochondria in PCCN.

## Conclusion

Our data illustrates that REST regulates neuron survival and is a critical neuroprotective factor in prion diseases and possibly other neurodegenerative disorders. The signally pathways for REST and associated molecules in neurodegenerative disorders are proposed (**Supplementary Figure S1**).

## Author Contributions

Conceived and designed the experiments: ZS. Performed the experiments: ZS, SS, WY, and HD. Analyzed the data: ZS. Wrote the paper: ZS. Edited the paper: ZS, SS, LY, XZ, and DZ. Contributed reagents/materials/analysis tools: DZ. All authors read and approved the final manuscript.

## Conflict of Interest Statement

The authors declare that the research was conducted in the absence of any commercial or financial relationships that could be construed as a potential conflict of interest.

## Supplementary Material

FIGURE S1 **Schematic signaling pathways for REST and Akt-mTOR and LRP6-Wnt-β-catenin pathways in prion diseases.** Exogenous REST (EX-REST) acts as a neuroprotective regulator in prion diseases state. Overexpressed REST contributes to neuronal survival by restoring the phosphorylation of Akt, mTOR and β-catenin proteins, inhibiting the formation of ROS and stabilizing the pro-survival protein CREB in PCCN. Mito, mitochondria. β-cat, β-catenin., phosphorylation. →: Direct stimulatory modification, →: Direct inhibitory modification, →: signaling becomes weak.
