# Supplementary figures and images for: Downregulation of the Repressor Element 1-Silencing Transcription Factor (REST) Is Associated with Akt-mTOR and Wnt-β-Catenin Signaling in Prion Diseases Models
Source: Front Mol Neurosci. 2017 May 3;10:128. doi: 10.3389/fnmol.2017.00128 (PMC5413570; doi:10.3389/fnmol.2017.00128)

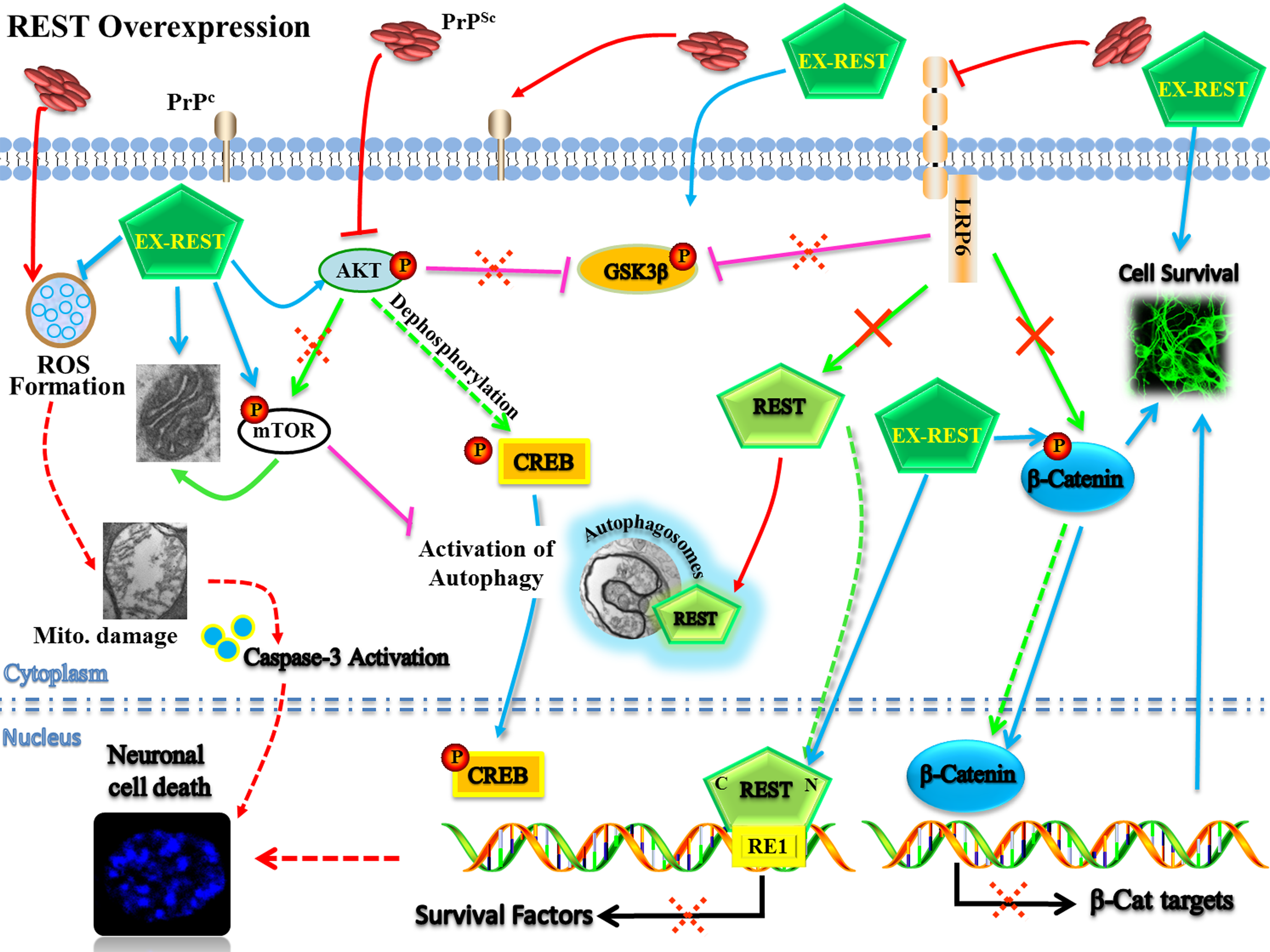

Supplement: FIGURE S1 — Schematic signaling pathways for REST and Akt-mTOR and LRP6-Wnt-β-catenin pathways in prion diseases. Exogenous REST (EX-REST) acts as a neuroprotective regulator in prion diseases state. Overexpressed REST contributes to neuronal survival by restoring the phosphorylation of Akt, mTOR and β-catenin proteins, inhibiting the formation of ROS and stabilizing the pro-survival protein CREB in PCCN. Mito, mitochondria. β-cat, β-catenin., phosphorylation. →: Direct stimulatory modification, →: Direct inhibitory modification, →: signaling becomes weak. [file Image_1.TIF]
